# Prognostic value of CA15-3, CA125, CEA in breast cancer patients undergoing chemotherapy

**DOI:** 10.5937/jomb0-55019

**Published:** 2025-10-28

**Authors:** Yue Zhang, Ying Ge, Yanhui Xu, Mengmeng Zhao

**Affiliations:** 1 The Second Norman Bethune Hospital of Jilin University, Department of Radiotherapy, Changchun, 130041, China

**Keywords:** exercise intervention under planned behaviour planning, breast tumours, chemotherapy, cancer-related fatigue, serum marker levels, intervencija vežbanja zasnovana na planiranju ponašanja, tumori dojke, hemoterapija, umor povezan sa rakom, nivoi serumskih markera

## Abstract

**Background:**

To explore the prognostic value of cancer antigen 15-3 (CA15-3), Carbohydrate antigen 125 (CA125), and carcino-embryonic antigen (CEA) in breast cancer patients undergoing chemotherapy.

**Methods:**

The data of 100 patients with breast cancer who received chemotherapy in a hospital from May 2022 to May 2024 were selected. Patients were divided into control and test groups based on different intervention methods and hospital stays. The control group receives routine nursing interventions. The test group implements exercise intervention under planned behaviour planning, with 50 cases in each group. Patients' exercise cognitive score, cancer-related fatigue, and serum tumour marker levels are compared before, 2 weeks, and 4 weeks of intervention.

**Results:**

Before the intervention, there was no difference in exercise cognition scores, cancer-related fatigue (CFS) scores, or serum tumour marker levels (P&gt;0.05). After 2 and 4 weeks of intervention, the exercise attitude, exercise values, exercise behaviour control, and exercise motivation in the test group exceeded the control group (P&lt;0.05). The behavioural, sensory, emotional, and cognitive fatigue scores in the test group were below the control group (P&lt;0.05). The CA15-3, CA125, and CEA in the test group were below the control group (P&lt;0.05).

**Conclusions:**

Exercise intervention under planned behaviour planning is conducive to improving the cognition of breast cancer chemotherapy patients on exercise knowledge, alleviating body fatigue, and reducing the serum markers.

## Abstract (sr)

**Uvod:**

Cilj je bio da se ispita prognostička vrednost antigena raka 15-3 (CA15-3), karbohidratičnog antigena 125 (CA125) i karcinoembrionskog antigena (CEA) kod pacijentkinja sa karcinomom dojke koje su na hemoterapiji.

**Metode:**

Analizirani su podaci 100 pacijentkinja sa karcinomom dojke koje su primale hemoterapiju u bolnici u periodu od maja 2022. do maja 2024. godine. Pacijentkinje su podeljene u kontrolnu i ispitivanu grupu na osnovu različitih metoda intervencije i dužine hospitalizacije. Kontrolna grupa je primala standardne medicinske intervencije, dok je u ispitivanoj grupi sprovedena intervencija vežbanja u skladu sa planiranjem ponašanja, pri čemu je svaka grupa imala po 50 pacijentkinja. Upoređivani su skorovi kognicije o vežbanju, umor povezan sa rakom i nivoi serumskih tumorskih markera pre intervencije, nakon 2 i 4 nedelje.

**Rezultati:**

Pre intervencije, nije bilo značajnih razlika u skorovima kognicije o vežbanju, skorovima umora povezanog sa rakom (CFS) i nivoima serumskih tumorskih markera (P&gt;0,05). Nakon 2 i 4 nedelje intervencije, stav prema vežbanju, vrednovanje vežbanja, kontrola ponašanja vezanog za vežbanje i motivacija za vežbanje bili su značajno viši u ispitivanoj grupi u poređenju sa kontrolnom grupom (P&lt;0,05). Skorovi fizičkog umora, senzornog umora, emo-cionalnog umora i kognitivnog umora bili su niži u ispiti-vanoj grupi u odnosu na kontrolnu grupu (P&lt;0,05). Nivoi CA15-3, CA125 i CEA u ispitivanoj grupi bili su niži u odnosu na kontrolnu grupu (P&lt;0,05).

**Zaključak:**

Intervencija vežbanja zasnovana na planiranju ponašanja doprinosi poboljšanju znanja pacijentkinja sa kar cinomom dojke na hemoterapiji o važnosti fizičke aktivnosti, ublažavanju telesnog umora i smanjenju nivoa serumskih tumorskih markera.

## Introduction

Cancer-related fatigue (CPF) is a persistent and debilitating condition commonly experienced by cancer patients, particularly those undergoing chemotherapy. Unlike ordinary fatigue, CPF is not relieved by rest and significantly reduces the quality of life [1] [2] [3] [4]. Breast cancer patients undergoing chemotherapy often experience CPF due to multiple contributing factors, including immune dysfunction [5] [6], inflammatory metabolic responses [7], and lifestyle habits [8]. Studies indicate that CPF is one of the most frequent side effects of cancer treatment, affecting up to 80% of patients, and its prevalence continues to rise [9]. In addition to worsening fatigue, chemotherapy leads to an increase in CA15-3 [2], CA125 [3], and CEA [4], which are essential serum tumour markers used for cancer diagnosis, progression monitoring, and treatment evaluation. Elevated levels of these biomarkers are strongly associated with disease severity, poor prognosis, and decreased survival rates [5].

Given the relationship between CPF and tumour marker elevation, interventions that mitigate fatigue while simultaneously reducing CA15-3, CA125, and CEA levels are increasingly being explored. Motor function training has been identified as a valuable strategy for improving CPF and regulating tumour markers [10]. Various exercise modalities, including Reiki therapy [11], yoga [12], and grip-strength exercises [13], have demonstrated some effectiveness in reducing CPF and improving serum markers. However, these methods are limited in scope, lack a structured theoretical foundation, and often fail to ensure long-term patient adherence. Furthermore, while sports training improves body function and reduces serum markers, its effectiveness is compromised when patients lack proper guidance, a standardised training plan, or adequate supervision. Many patients also experience difficulty adhering to exercise regimens, leading to reduced compliance and suboptimal outcomes. Additionally, unsupervised self-exercise can increase injury risk due to improper training intensity or execution [14] [15].

To address these limitations, exercise intervention under planned behaviour planning (PBP) has been proposed as a structured, evidence-based approach to improving both exercise adherence and physiological outcomes in breast cancer patients. PBP is based on Ajzen’s Planned Behaviour Theory, which identifies barriers to exercise, predicts behavioural intentions, and enhances individual decision-making processes related to physical activity [16]. This intervention aims to improve exercise cognition, motivation, and compliance, reducing CPF and improving tumour marker levels [17]. In prostate cancer patients undergoing androgen deprivation therapy, structured exercise training has been shown to effectively reduce fatigue and enhance overall quality of life [18]. Similarly, low-frequency exercise training in breast cancer patients has been reported to improve cardiovascular function, alleviate treatment-related symptoms, and significantly reduce CA15-3, CA125, and CEA levels [19].

This study focuses on breast cancer patients undergoing chemotherapy and evaluates the impact of exercise intervention under planned behaviour planning on serum tumour markers and cancer-related fatigue. By enhancing exercise adherence, improving physical resilience, and lowering biomarker levels, this intervention offers a promising strategy for optimising treatment outcomes, improving patient prognosis, and enhancing overall well-being.

## Materials and methods

### Study design and implementation method

This study was a prospective, controlled clinical trial designed to evaluate the effects of exercise intervention under planned behaviour planning on chemotherapy-induced fatigue (CPF), sports cognition, and serum tumour markers (CA15-3, CA125, and CEA) in breast cancer patients undergoing chemotherapy.

A total of 100 breast cancer patients receiving chemotherapy from May 2022 to May 2024 were included in the study. Patients were randomly assigned to one of two groups:

- Control Group (CG) (n=50): Received routine nursing interventions, including general chemotherapy education, dietary guidance, observation of adverse reactions, and general exercise recommendations (walking, brisk walking, and Tai Chi).
- Test Group (TG) (n=50): Received four-week routine nursing interventions and exercise under planned behaviour planning.

This exercise intervention was designed using behavioural psychology and health promotion principles to improve patients’ exercise awareness, motivation, and adherence. It involved behaviour attitude modification, subjective norm reinforcement, and perceived behavioural control techniques to enhance exercise compliance and improve physical outcomes.

### Research materials

A total of 100 breast cancer patients undergoing chemotherapy were included. Patients were divided into two groups (CG and TG), with 50 cases each. There were no statistically significant differences in age, disease type, Body Mass Index (BMI), cultural background, and TNM staging between the groups (P>0.05), as displayed in Table 1.

**Table 1:** Comparison of baseline information between two groups (x̄±s, %).

Groups	n	Age (years)	Disease type			BMI (kg/m^2^)	
			Infiltrating ductal<br>carcinoma	Intraductal<br>carcinoma	Others		
Control group	53	45.15±10.26	18(33.96)	23(46.94)	12(24.49)	20.15±2.16	
Test group	53	45.98±10.06	20(40.82)	25(41.02)	18(36.73)	20.67±2.09	
* χ^2^/t *	/	0.404	0.531			1.211	
* P *	/	0.687	0.767			0.229	
Groups	n	Cultural background			TNM stage		
		Junior high<br>school and<br>below	High<br>school	College degree<br>or above	I	II	III
Control group	53	3(5.60)	28(57.14)	22(41.51)	18(33.96)	26(49.06)	9(16.98)
Test group	53	5(10.20)	31(63.27)	17(32.08)	15(28.30)	22(41.51)	16(30.19)
* χ2/t *	/	1.294			2.566		
* P *	/	0.524			0.277		

### Inclusion criteria

1. Confirmed breast cancer diagnosis via histopathological examination, meeting the 2018 Fourth ESO-ESMO Advanced Breast Cancer International Consensus Guidelines [20].
2. Patients undergoing first surgical treatment and adjuvant chemotherapy in the hospital.
3. Functional limbs, allowing for participation in exercise training.
4. Cognitively intact, with normal reading, writing, and comprehension abilities.

### Exclusion criteria

1. Patients unaware of their cancer diagnosis and unable to cooperate with treatment and nursing interventions.
2. Patients receiving concurrent radiation therapy.
3. Patients with coagulation dysfunction or a tendency to bleed.
4. Patients with severe distant metastasis.
5. Patients with other malignancies, such as liver cancer, gastric cancer, or oesophageal cancer.

### Research methods

Control Group (CG): Routine nursing interventions

Patients in the control group received routine nursing care, including:

- Chemotherapy education: Nurses explained chemotherapy drugs, side effects, and necessary precautions to patients and their families.
- Dietary guidance: Patients were instructed to follow a light and easily digestible diet, avoid eating 2 hours before chemotherapy, and monitor for adverse reactions.
- General exercise recommendations: Patients were advised to perform light exercises, such as walking, brisk walking, and Tai Chi, for 15–30 minutes per session with an optional increase in intensity based on tolerance.
- Post-discharge care: Patients received a health manual for home care and underwent regular follow-ups based on their scheduled medical reviews.

The intervention lasted for 4 weeks.

Test Group (TG): Exercise intervention under planned behaviour planning

The intervention in the test group was based on planned behaviour theory and included three core strategies:

### (A) Identifying barriers to exercise

- Patients participated in semi-structured interviews to discuss the factors affecting their exercise adherence.
- Patients were asked key questions such as:
- ° How do you feel about exercising during chemotherapy?
- ° Will you continue exercising during home recovery?
- ° How do family and friends influence your exercise habits?
- Patients were divided into 5 discussion groups (10 per group) to share experiences and challenges.
- Medical staff analysed these responses to develop a personalised intervention plan.

### (B) Implementing exercise intervention under planned behaviour planning

1. Behavioural attitude modification

- ° Patients were educated about the benefits of exercise, risks of inactivity, safety measures, and precautions using graphic manuals and multimedia videos.
- ° Patients were encouraged to ask questions, and misconceptions about exercise were corrected.
- ° Enhancing subjective norms (motivation for exercise).
- ° One-on-one consultations were provided for patients needing individualised exercise guidance.
- ° Family members were encouraged to support and participate in exercise training.
- ° Peer education: Patients with prior successful exercise experiences shared their exercise strategies and recovery journeys.

2. Perceived behavioural control (overcoming exercise barriers)

- ° Weekly group education sessions covered chemotherapy, disease progression, and exercise training to highlight the interconnections among these factors.

Exercise techniques included:

Progressive muscle relaxation training: Patients tensed and relaxed different muscle groups, focusing on each for 5 seconds before moving to the next.

Aerobic exercise training: Patients were guided on performing fast walking and jogging for 30–45 minutes per session, 3–5 times per week, based on their comfort level.

The intervention lasted for 4 weeks.

### Observation indicators and evaluation

Baseline information was collected from medical records, including the patient’s age, disease type, Body Mass Index (BMI), cultural background, and TNM staging.

The sports cognition level was assessed using a self-designed exercise cognition questionnaire based on the Montreal Cognitive Assessment (MOCA). This questionnaire evaluated four key dimensions: exercise attitude, exercise values, exercise behaviour control, and exercise motivation. The scoring system consisted of 20 questions rated on a four-point scale, with a total possible score ranging from 0 to 100. Higher scores indicated better exercise cognition. The reliability of the questionnaire was confirmed with a Cronbach’s of 0.902, demonstrating good validity and consistency.

### Chemotherapy-induced fatigue (CPF)

Chemotherapy-induced fatigue (CPF) was measured using the Cancer Fatigue Scale (CFS), which assessed three dimensions: physical, cognitive, and emotional. The bodily fatigue component included seven items, while cognitive and emotional fatigue was measured with four items each. The scoring system used a 1-to-5 scale, with a total possible score of 0 to 60. Higher scores indicated more severe CPF. The scale demonstrated high reliability and validity, with a Cronbach’s of 0.864.

### Serum marker levels

The study measured specific tumour markers, including cancer antigen 15-3 (CA15-3), carbohydrate antigen-125 (CA125), and carcinoembryonic antigen (CEA). For the analysis, 5 mL of venous blood was drawn after an overnight fast. The collected blood samples were centrifuged at 3000 rpm for 10 minutes, and the supernatant was analysed using a fully automated chemiluminescence instrument. Electrochemical luminescence was used for marker detection.

### Statistical data analysis

Data analysis was conducted using SPSS 25.0 software. Categorical data were expressed as frequency (n) and percentage (%) and analysed using the χ^2^ test. Continuous variables were presented as mean ± standard deviation (x̄±s). Independent sample t-tests were used to compare differences between groups, while paired sample t-tests were applied for within-group comparisons. A significance level of P<0.05 was considered statistically significant.

## Results

### Demographic data

The study included a total of 106 participants, divided equally into a control group and a test group, with 53 individuals in each. The average age of participants was approximately 45 years, with no statistically significant difference between the two groups (P>0.05). The distribution of disease types included infiltrating ductal carcinoma, intraductal carcinoma, and other types, with similar proportions between groups. BMI values were comparable, averaging around 20.5 kg/m^2^ in both groups. Participants’ cultural backgrounds varied, with education levels ranging from junior high school and below to college degrees and above. The TNM staging was distributed across stages I, II, and III, with no significant differences between the two groups. These baseline characteristics confirmed that the two groups were comparable before intervention (P>0.05).

### Comparison of two sets of baseline information data

There was no statistically significant difference in age, disease type, BMI, cultural background, and TNM staging between the two groups (*P*>0.05), as shown in Table 1.

### Comparison of exercise cognition scores before intervention, 2 weeks and 4 weeks after intervention

Before the intervention, exercise cognition scores (exercise attitude, values, behaviour control, and motivation) showed no significant differences between groups (P>0.05).

After 2 and 4 weeks of intervention, all exercise cognition parameters increased significantly, with the TG showing greater improvements than the CG (P<0.05).

Exercise Attitude: Increased more in TG (16.57±3.96 vs. 14.89±3.26 in CG, P<0.05).

Exercise Values: Higher in TG after 4 weeks (16.07±2.93 vs. 14.69±2.65, P<0.05).

Exercise Behavior Control: Greater improvement in TG (16.38±2.98 vs. 14.69±2.51, P<0.05).

Exercise Motivation: Notably enhanced in TG (15.29±3.47 vs. 13.59±2.54, P<0.05).

**Figure 1:**
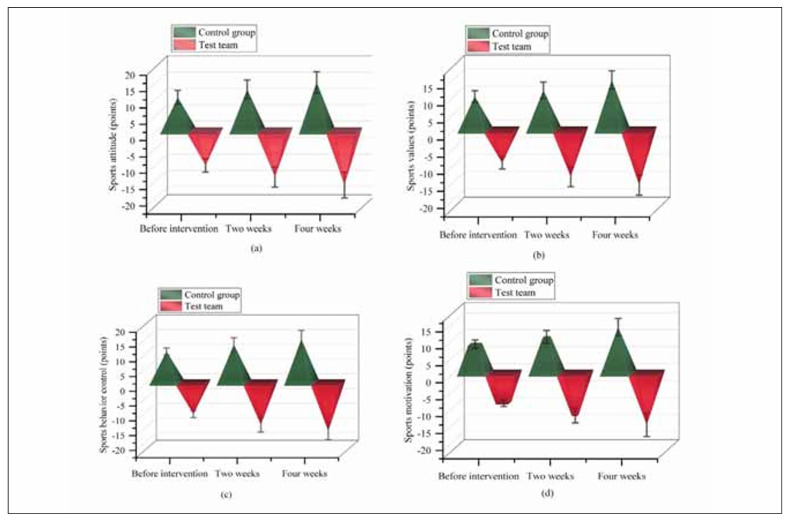
Comparison of exercise cognition scores at different periods using a 3D side-by-side pyramid chart.

These findings suggest that exercise intervention under planned behaviour planning (PBP) significantly improves exercise awareness and adherence. The detailed results are provided in Table 2 and visualised in Figure 1.

**Table 2:** Comparison of exercise cognition scores before intervention, 2 and 4 weeks after intervention (x̄±s, %). Note: Compared with before intervention in this group, *P*<0.05. Compared with the 2-week intervention in this group, *P*<0.05. Compared with the 4-week intervention in this group, *P*<0.05.

Groups	n	Exercise attitude			Exercise values		
		Before<br>intervention	Intervention<br>for 2 weeks	Intervention<br>for 4 weeks	Before<br>intervention	Intervention<br>for 2 weeks	Intervention<br>for 4 weeks
Control group	53	10.26±2.16	12.69±2.86*	14.89±3.26*^#^	9.88±1.68	11.69±2.34*	14.69±2.65*^#^
Test group	53	10.57±2.03	14.11±3.10*	16.57±3.96	9.62±1.80	13.64±2.91*	16.07±2.93*^#^
* t *	/	0.761	2.451	2.384	0.769	3.802	2.543
* P *	/	0.448	0.016	0.019	0.444	0.000	0.013
Groups	n	Exercise behaviour control			Exercise motivation		
		Before<br>intervention	Intervention<br>for 2 weeks	Intervention<br>for 4 weeks	Before<br>intervention	Intervention<br>for 2 weeks	Intervention<br>for 4 weeks
Control group	53	10.55±1.18	12.85±2.34*	14.69±2.51*^#^	8.56±1.22	10.69±1.89*	13.59±2.54*^#^
Test group	53	10.71±1.19	14.26±2.45*	16.38±2.98*^#^	8.81±1.06	12.54±2.16*	15.29±3.47*^#^
* t *	/	0.695	3.030	3.158	1.126	4.693	2.878
* P *	/	0.489	0.003	0.002	0.263	0.000	0.005

Compared with the CG receiving the routine nursing intervention, there was no significant difference in exercise attitude, exercise values, exercise behaviour control, and exercise motivation scores before intervention (*P*>0.05). After the intervention, all indicators in the TG exceeded the CG (*P*<0.05), as displayed in Figure 1.

### Comparison of CFS scale scores before intervention, 2 and 4 weeks after intervention

Before intervention, there was no significant difference in the scores on the CFS scale (*P*>0.05). After 2 weeks and 4 weeks of intervention, both groups showed a decrease in behavioural fatigue, sensory fatigue, emotional fatigue, cognitive fatigue, and total scores. The TG was below the CG (*P*<0.05), as displayed in Table 3.

**Table 3:** Comparison of CFS scale scores before intervention, 2 weeks and 4 weeks after intervention (x̄±s, scores). Note: Compared with before intervention in this group, P<0.05. Compared with the 2-week intervention in this group, P<0.05. Compared with the 4-week intervention in this group, P<0.05. CFS: Cancer Fatigue Scale.

Groups	n	Physical fatigue			Cognitive fatigue		
		Before<br>intervention	Intervention <br>for 2 weeks	Intervention <br>for 4 weeks	Before <br>intervention	Intervention <br>for 2 weeks	Intervention <br>for 4 weeks
Control group	53	28.12±4.34	23.57±1.10*	14.06±0.87*^#^	18.56±2.41	14.38±1.16*	10.16±0.68*^#^
Test group	53	28.36±4.19	20.21±0.98*	10.36±0.72*^#^	18.67±2.39	11.29±1.00*	7.39±0.37*^#^
* t *	/	0.290	16.604	23.852	0.236	14.688	26.049
* P *	/	0.773	0.000	0.000	0.814	0.000	0.000
Groups	n	Emotional fatigue			Total score		
		Before <br>intervention	Intervention <br>for 2 weeks	Intervention <br>for 4 weeks	Before <br>intervention	Intervention <br>for 2 weeks	Intervention <br>for 4 weeks
Control group	53	17.89±2.10	15.23±1.67*	12.20±0.89*^#^	64.57±8.85	53.18±3.93	36.42±2.44
Test group	53	17.95±2.03	13.11±1.89*	10.09±0.36*^#^	64.98±8.61	44.61±3.87	27.84±1.45
* t *	/	0.150	6.119	16.000	0.242	11.312	22.007
* P *	/	0.881	0.000	0.000	0.810	0.000	0.000

**Figure 2:**
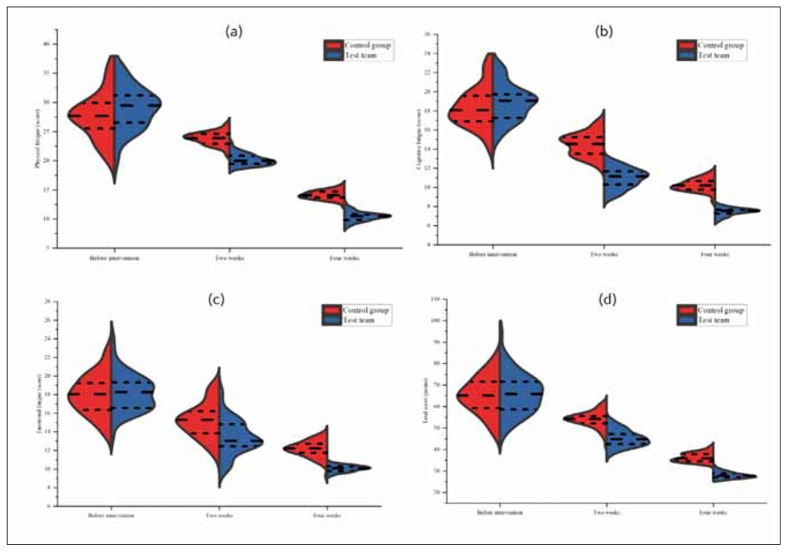
The scores of CFS scales in two different time segments on the violin chart.<br>Note: Figure 2 (a) to (d) represent physical fatigue, cognitive fatigue, emotional fatigue, and the total score of the CFS scale, respectively. Red represents the CG. The blue colour represents the TG. Long-spaced horizontal lines indicate intervention for 2 weeks. The short spacing of the horizontal line at the bottom indicates that it was before the intervention. Short spacing horizontal line at the bottom indicates intervention for 4 weeks.

The vertical axis was displayed as a reference ruler for comparison. There was no statistically significant difference in physical fatigue, cognitive fatigue, emotional fatigue, and total CFS scores between the two groups before intervention (*P*>0.05). After intervention for 2 and 4 weeks, all indicators in the TG were below the CG (*P*<0.05), as displayed in Figure 2.

### Comparison of CA15-3, CA125, and CEA between two groups before intervention, 2 and 4 weeks after intervention

Before the intervention, the two groups had no statistically significant difference in serum marker levels (*P*>0.05). After 2 weeks and 4 weeks of intervention, both groups’ CA15-3, CA125, and CEA decreased. The TG was below the CG (*P*<0.05), as displayed in Table 4.

**Table 4:** Comparison of CA15-3, CA125, and CEA before intervention, 2 and 4 weeks after intervention (x̄±s). Note: Compared with before intervention in this group, *P*<0.05. Compared with the 2-week intervention in this group, *P*<0.05. Compared with the 4-week intervention in this group, *P*<0.05. CA15-3: Cancer antigen 15-3. CA125: Carbohydrate antigen. CEA: Carcinoembryonic Antigen.

Groups	n	CA15-3 (ng/L)			CA125 (ng/L)		
		Before<br>intervention	Intervention <br>for 2 weeks	Intervention <br>for 4 weeks	Before <br>intervention	Intervention <br>for 2 weeks	Intervention <br>for 4 weeks
Control group	53	59.89±11.37	54.12±10.23*	48.97±9.56*^#^	72.98±20.15	63.41±19.57*	53.28±18.56*
Test group	53	59.67±10.59	49.06±10.05*	44.26±8.37*^#^	72.56±20.81	54.22±18.16*	41.12±17.20*
* t *	/	0.103	2.569	2.699	0.106	2.506	3.498
*P *	/	0.918	0.012	0.008	0.916	0.014	0.001
Groups	n	CEA (ng/L)					
		Before intervention		Intervention for 2 weeks		Intervention for 4 weeks	
Control group	53	11.59±2.64		10.31±2.07*		7.68±1.93*#	
Test group	53	11.71±2.39		8.95±1.86*		4.83±1.52*#	
* t *	/	0.245		3.558		8.446	
* P *	/	0.807		0.001		0.000	

**Figure 3:**
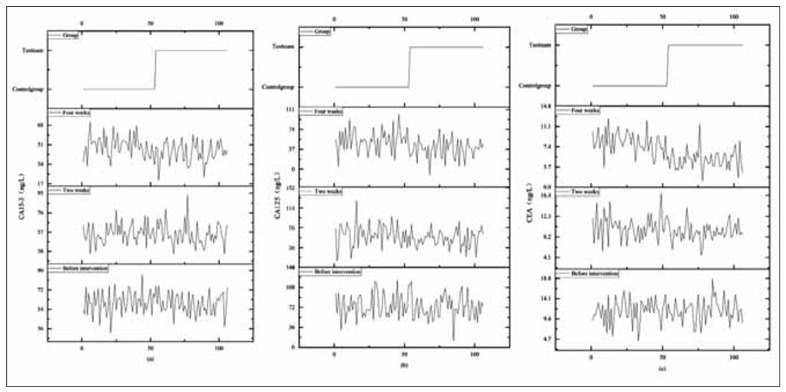
Stacked chart of comparison of CA15-3, CA125, and CEA Levels between two groups at different period.<br>Note: Figures (a) to (c) represent CA15-3, CA125, and CEA, respectively. One side of the low-level straight line is the CG, while the other side of the high-level straight line is the TG. The vertical axis interval line from bottom to top is before intervention, 2 weeks after intervention, and 4 weeks after intervention. The stacked lines in the graph represent the comparison of various indicators between the two groups at different time periods.

The vertical axis is used as a reference ruler. They had no statistically significant difference in the CA15-3, CA125, and CEA before the intervention implementation (*P*>0.05). After intervention for 2 and 4 weeks, the various indicators in the TG were below the CG (*P*<0.05), as displayed in Figure 3.

## Discussion

Chemotherapy for breast cancer is a long-term process. The condition of cancer itself, chemotherapy, and daily exercise habits are all important factors that lead to CPF. At the same time, it also reduces the effectiveness of disease treatment. It is the fundamental reason for the increase in CA15-3, CA125, and CEA [20] [21]. Patients with breast cancer have low awareness of sports, which will reduce their compliance with sports training. CPF cannot be improved [22]. Breast cancer patients are also affected by training methods, training norms, training continuity and training intensity. The effect of sports training is not ideal [23]. Therefore, the current situation of CPF and exercise training for breast cancer chemotherapy patients is comprehensively guided and intervened.

Exercise intervention under planned behaviour planning was a method in behavioural psychology and health promotion. It aims to help breast cancer patients develop clear exercise plans to improve their sports cognition [24], enhancing confidence and motivation in sports [25]. Exercise intervention under planned behaviour planning can provide mental health support for breast cancer patients [26]. Moderate and regular exercise can stimulate the brain to release neurotransmitters such as dopamine [27], endorphins [28], and serotonin [29]. The secretion of these substances can improve the patient’s mood and alleviate anxiety and depression. Exercise intervention under planned behaviour planning can enhance the adaptability of breast cancer patients, improve their physical strength and endurance, and better cope with the discomfort caused by chemotherapy [30]. The application of exercise intervention under planned behaviour planning in breast cancer chemotherapy had many benefits, including improving patients’ sports cognition, providing mental health support, and improving physical strength and endurance. By improving the overall status of breast cancer patients, their CPF and the CA15-3, CA125 and CEA were improved.

Min and colleagues applied the Theory of Planned Behaviour (TPB) to assess exercise intentions in Korean breast cancer survivors, finding that attitudes, subjective norms, and perceived behavioural control (PBC) significantly influenced physical activity. Our study on exercise intervention under planned behaviour planning (PBP) aligns with these findings, demonstrating that structured exercise improves adherence, reduces cancer-related fatigue (CPF), and lowers tumour markers (CA15-3, CA125, CEA) in chemotherapy patients. It was focused on psychosocial factors, our research highlights both behavioural and physiological benefits, suggesting that integrating TPB-based exercise interventions can enhance patient outcomes. Future studies should explore long-term adherence and biomarker regulation to optimise recovery strategies.

The results of this study align with previous research, confirming that exercise intervention under planned behaviour planning (PBP) effectively enhances exercise cognition, reduces cancer-related fatigue (CPF), and lowers serum tumour marker levels in breast cancer patients undergoing chemotherapy. The improvement in exercise attitude, values, behaviour control, and motivation observed in the test group (TG) supports existing findings that structured exercise programs help patients develop clear exercise plans, boosting confidence and adherence to physical activity.

The significant reduction in physical, cognitive, and emotional fatigue further validates the role of exercise in alleviating chemotherapy-related side effects. Prior studies suggest that moderate, regular exercise stimulates the release of neurotransmitters like dopamine, endorphins, and serotonin, which help improve mood, reduce anxiety, and combat fatigue. Our findings reinforce this, as patients in the TG experienced greater relief from fatigue symptoms compared to the control group (CG). Additionally, the decline in CA15-3, CA125, and CEA levels in the TG indicates that structured exercise may contribute to better disease management and prognosis. Similar studies have highlighted that exercise reduces inflammatory responses and enhances immune function, positively influencing chemotherapy outcomes. The observed decrease in tumour markers suggests that exercise interventions can support ongoing treatment by mitigating cancer progression.

After 2 and 4 weeks of exercise intervention under the planned behaviour planning, the TG patients had higher exercise attitude, exercise values, exercise behaviour control, and exercise motivation cognition than the CG who implemented routine training. The reasons are as follows: Exercise intervention under planned behaviour planning provides patients with health information related to exercise through graphic manuals and multimedia videos. Strengthening patients’ positive attitudes towards exercise improved their cognitive exercise training knowledge [31]. For patients with still low cognitive levels, one-on-one guidance was implemented. Patients were encouraged to communicate, which helped them change their subjective norms, strengthened their expectations and social pressure to participate in sports, and enhanced their understanding of sports knowledge [32]. The patient’s perceptual and behavioural were better controlled, helping them identify obstacles to implementing exercise plans, providing timely strategies to overcome barriers and comprehensive psychological support, and enhancing their understanding of exercise knowledge [33]. The positive attitude and intention to exercise intervention of colon cancer patients were analysed. If patients believed exercise was beneficial for their health, mental health, and CPF, they were more likely to exhibit positive exercise intentions. That is, attitudes could affect patients’ behavioural intentions [33]. Social support and others’ expectations can affect nasopharyngeal cancer patients’ exercise intention and behaviour. If patients could feel the support of family, friends, and medical teams, they would be likelier to show a more positive intention to exercise [34]. However, among survivors of multiple myeloma cancer, the impact of planned behaviour theory on exercise was analysed. Patients’ compliance with exercise was improved by changing their subjective norms, perceived behavioural control, and emotional attitudes towards exercise. The patient’s cognition for exercise knowledge was not analysed [35]. The planned behaviour theory examined the determinants of bladder cancer survivors’ exercise. Adjuvant therapy, cancer invasion degree, and age could all affect patients’ cognitive level for exercise knowledge [36].

After 2 and 4 weeks of exercise intervention under planned behaviour planning, the TG showed lower behavioural, sensory, emotional, cognitive, and total fatigue scores than the CG. The main reasons were as follows. Exercise intervention under planned behaviour planning was based on improving patients’ understanding of exercise knowledge and strengthening their confidence in exercise. After the intervention, patients improved their understanding of sports knowledge and enthusiasm for exercise training. They underwent regular and effective exercise training based on their own tolerance to exercise, avoiding injuries caused by high-intensity training and ensuring the continuity and effectiveness of exercise training. The guidance of nursing staff and peer communication could fundamentally reduce the adverse factors affecting the exercise of breast cancer patients, improve the effect of exercise training, and improve the CPF [37]. Patients with physical training, especially those with breast cancer or prostate cancer, had relatively mild CPF [38]. After long-term and regular exercise training, the body’s functions gradually improved, alleviating the CPF. However, patients with locally advanced or metastatic cancer who were severely fatigued could only rely on treatment to alleviate cancer fatigue due to limitations in their condition, exercise intensity, and exercise time. Exercise could not alleviate CPF.

CA15-3 was a specific marker index of breast cancer [39]. It was used in the treatment of breast cancer and the monitoring of recurrence. If CA15-3 in the blood increased, it indicated the possibility of tumour cell activity and recurrence. Further inspection was needed to determine the situation. CA125 was a specific biomarker for ovarian cancer [40]. The blood level of patients with breast cancer, pancreatic cancer and rectal cancer also increased, which was mainly used for auxiliary diagnosis, monitoring and evaluation of tumour recurrence. CEA was a non-specific marker [41]. It was produced in various cancer cells, which was used for early diagnosis and disease monitoring of cancer cells.

After 2 and 4 weeks of exercise intervention under planned behaviour planning, CA15-3, CA125 and CEA of breast cancer patients were below the CG. The reasons were as follows. Exercise intervention under planned behaviour planning could improve the immune capacity of patients with breast cancer undergoing chemotherapy and reduce the occurrence of complications. Effective exercise could stimulate the secretion of neurotransmitters in the body, helping patients maintain a good psychological state. Exercise intervention under planned behaviour planning was applied to breast cancer patients to reduce the impact of adverse factors on treatment, ensure the smooth implementation of treatment, and improve the treatment effect of diseases, thus alleviating the state of illness and reducing CA15-3, CA125 and CEA. Exercise intervention could reduce the white blood cell count [42] and C-reactive protein (CRP) levels [43] of chemotherapy patients with breast cancer. The main intervention implementation could change the patient’s behavioural intention, improve their exercise level, reduce the body’s inflammatory response and improve serum marker levels. Exercise intervention could reduce the weight of breast cancer patients and improve insulin sensitivity [44]. It also reduces the body’s estrogen levels [45]. The overall endocrine function of the body was improved, providing basic rehabilitation conditions for disease recovery, thereby reducing serum biomarker levels. After one week of training, gastric cancer patients were evaluated for serum indicator levels. No significant improvement was observed. This was mainly due to the impact of training frequency, intensity, and time. Exercise did not achieve the desired effect. The overall physical fitness decreased, resulting in differences in serum marker levels [46] [47].

To sum up, breast cancer chemotherapy patients implement exercise intervention under planned behaviour planning. By intervening in their attitude, subjective view and perceived behaviour, they can reduce the influencing factors of sports training, strengthen their confidence and intention in sports training, and improve their enthusiasm for sports training. Their body quality and CPF are alleviated, improving the treatment effect. The levels of CA15-3, CA125, and CEA are improved. Although the research has achieved good results, the selected single-centre sample has led to different results. There is no long-term observation and evaluation of the patient’s exercise rehabilitation compliance and quality of life indicators after treatment, leading to limitations in the research results. Therefore, based on this study, multi-centre research subjects should be selected to reduce the differences in single-centre studies. At the same time, the long-term evaluation indicators of breast cancer patients will be increased to understand the patients’ long-term compliance with exercise training and quality of life. It is expected to make the research content more comprehensive and perfect, providing important references for clinical follow-up research.

## Conclusion

This study evaluated the impact of exercise intervention under planned behaviour planning on cancer-related fatigue (CPF) and serum tumour markers in breast cancer patients undergoing chemotherapy. Results showed that the intervention improved exercise cognition, reduced fatigue, and significantly lowered CA15-3, CA125, and CEA levels. These findings suggest that structured exercise can enhance patient well-being and treatment outcomes. However, further multi-centre studies with long-term follow-ups are needed to validate these results and optimise rehabilitation strategies.

## Dodatak

### Conflict of interest statement

All the authors declare that they have no conflict of interest in this work.
